# The Relationship Between Resting State Network Connectivity and Individual Differences in Executive Functions

**DOI:** 10.3389/fpsyg.2018.01600

**Published:** 2018-09-05

**Authors:** Andrew E. Reineberg, Daniel E. Gustavson, Chelsie Benca, Marie T. Banich, Naomi P. Friedman

**Affiliations:** ^1^Department of Psychology and Neuroscience, University of Colorado Boulder, Boulder, CO, United States; ^2^Institute for Behavioral Genetics, University of Colorado Boulder, Boulder, CO, United States; ^3^Department of Psychiatry, University of California, San Diego, San Diego, CA, United States; ^4^Department of Psychology, Emory University, Atlanta, GA, United States; ^5^Institute of Cognitive Science, University of Colorado Boulder, Boulder, CO, United States

**Keywords:** executive function, functional connectivity, networks, resting-state, fMRI

## Abstract

The brain is organized into a number of large networks based on shared function, for example, high-level cognitive functions (frontoparietal network), attentional capabilities (dorsal and ventral attention networks), and internal mentation (default network). The correlations of these networks during resting-state fMRI scans varies across individuals and is an indicator of individual differences in ability. Prior work shows higher cognitive functioning (as measured by working memory and attention tasks) is associated with stronger negative correlations between frontoparietal/attention and default networks, suggesting that increased ability may depend upon the diverging activation of networks with contrasting function. However, these prior studies lack specificity with regard to the higher-level cognitive functions involved, particularly with regards to separable components of executive function (EF). Here we decompose EF into three factors from the unity/diversity model of EFs: Common EF, Shifting-specific EF, and Updating-specific EF, measuring each via factor scores derived from a battery of behavioral tasks completed by 250 adult participants (age 28) at the time of a resting-state scan. We found the hypothesized segregated pattern only for Shifting-specific EF. Specifically, after accounting for one’s general EF ability (Common EF), individuals better able to fluidly switch between task sets have a stronger negative correlation between the ventral attention network and the default network. We also report non-predicted novel findings in that individuals with higher Shifting-specific abilities exhibited more positive connectivity between frontoparietal and visual networks, while those individuals with higher Common EF exhibited increased connectivity between sensory and default networks. Overall, these results reveal a new degree of specificity with regard to connectivity/EF relationships.

## Introduction

Executive functions (EFs) are a set of higher-level cognitive abilities that contribute to the maintenance, implementation, and modification of goals (Banich, 2009; Friedman and Miyake, 2017). Classically, EFs have been linked to frontal lobe function based on both studies of individuals with localized lesions (Stuss and Alexander, 2000; Alvarez and Emory, 2006) and on task-based functional magnetic resonance imaging studies (fMRI; Wager and Smith, 2003; Wager et al., 2004). Recent work has begun to examine other potential neural correlates of EFs, in particular, connectivity between large scale brain systems. Brain systems can be studied in many contexts. For example, networks of brain regions that are involved in similar processes (functional networks) are observed in task-based fMRI studies when specific cognitive constructs are targeted with subtraction-based methods and in fMRI studies of resting-state functional connectivity. Resting-state functional connectivity refers to the observation that regions of related function have similar time courses of low frequency BOLD signal when individuals are asked to merely relax inside an fMRI scanner. The resting state is a particularly interesting context because it is mostly free of instruction-related demands on participants and provides a measure of coordination (time course correlation) between functional networks that is highly stable (Shehzad et al., 2009; Choe et al., 2015).

While an intact frontal system may be necessary for performing EF tasks, high functioning may depend upon the segregation of EF-related mechanisms from contrasting mechanisms such as those related to internal mentation. Prior work in the clinical domain has found that depression is associated with co-activation of resting-state brain systems responsible for cognitive control and internal mentation (Kaiser et al., 2016), which is one possible explanation for EF-related deficits that are frequently observed in individuals suffering from depression and/or other forms of psychopathology (Snyder et al., 2015). In addition, some preliminary work in neurologically normal individuals has shown that variation in connectivity between networks is linked to individual differences in cognitive ability. For example, altered connectivity between networks responsible for externally- versus internally directed attention has been observed in individuals with high versus low working memory ability as measured by both sequencing and span tasks (Keller et al., 2015) and across individuals with variations in attentional control ability (Kelly et al., 2008). The tasks used in these studies index some cognitive abilities specific to working memory and attentional mechanisms, respectively, but also measure common mechanisms such as the ability to learn/maintain complex rules or insulate task goals from competing personal thoughts, abilities shared across many EF tasks. Thus, the nature of these previously reported brain/behavior relationships are imprecise due to task impurity, leading to the question of whether network connectivity is a neural correlate of processes common to many cognitive tasks or those specific to a particular task or operation.

The current study utilizes a multitask EF battery to more specifically investigate the link between network connectivity and EFs. We utilize the Unity/Diversity model of EF, an influential framework that re-parameterizes variance in three commonly studied EF processes (prepotent response inhibition, mental set shifting, and working memory updating) into three orthogonal latent factors (Miyake and Friedman, 2012; Friedman and Miyake, 2017). The first factor, Common EF, accounts for performance on all EF tasks, and is thought to reflect the ability to actively maintain and implement a task goal or attentional set. Two orthogonal diversity factors predict additional variance in the shifting and updating tasks. Shifting-specific EF is thought to reflect the speed with which one can clear goals that are no longer relevant, beyond those goal-management processes recruited in Common EF. Similarly, Updating-specific EF reflects working memory operations that are not captured by Common EF, such as gating and possibly episodic retrieval. There is no evidence for an inhibition-specific factor, suggesting that individual differences in response inhibition are captured by Common EF (Friedman and Miyake, 2017). Hence, this framework captures both unity (Common EF) and diversity (Shifting-specific and Updating-specific) of EFs.

Although prior work has focused on the correlation of specific regions of interest within the functional networks implicated in externally and internally directed attention, we utilize a whole-cortex network approach so as to not limit ourselves to specific, subjectively chosen functional regions of interest. This approach also affords the potential to reveal novel network connectivity/EF relationships. There is overwhelming evidence of functional networks from parcellation studies of brain activity during resting-state scans (Power et al., 2011; Yeo et al., 2011). For the current analysis, we chose a popular low-dimensionality solution as determined by a clustering analysis of resting-state scans from over 1000 individuals; this solution describes seven networks: visual, sensory/somatomotor, dorsal attention, ventral attention, salience, default, and frontoparietal networks (Yeo et al., 2011; the authors also provide a 17-network solution we utilize to provide more detail on EF-related connections).

Within this framework, visual and sensory-somatomotor networks are well-characterized and contain regions located in close proximity to V1 and the sensory/motor strips, respectively. The limbic network contains predominantly orbitofrontal cortex (Mega et al., 1997), which is involved in affect, valuation, and decision-making. The remaining networks of the seven-network parcellation can broadly be categorized as *task positive* or *task negative* based on whether or not their activation typically increases or decreases, respectively, during difficult, externally directed cognitive control tasks when compared to baseline. In the parcellation provided by Yeo and colleagues, there are three task-positive networks — the frontoparietal, dorsal attention, and ventral attention networks — that are implicated in the various levels of control needed to perform directed tasks. The dorsal attention network is involved in top-down biasing of attention during goal pursuit, whereas the ventral attention network is involved bottom up attentional processes such as reorienting or filtering of attention toward sensory information in the environment that may be goal-related (Vossel et al., 2014). The frontoparietal network is implicated in higher-level functions such as fine adjustment of current behavior in response to changes in task demands (Dosenbach et al., 2008). The task-negative network is the default network, which is a set of midline frontal, posterior cingulate, and middle temporal areas implicated in a family of self-related processes such as imagination and reminiscence (Andrews-Hanna, 2011). The typical decrease in BOLD signal of the default network during difficult externally directed tasks is explained as a decreased focus on the internal world and redirection of attention to the demanding task (Fox et al., 2005).

We examined the hypothesis that individual differences in EFs are associated with individual differences in correlation strength between task-positive and task-negative networks. Prior studies have found evidence of hypoconnectivity (decreased positive/increased negative connectivity) in individuals with higher working memory span and sequencing ability (e.g., Keller et al., 2015). However, due to ambiguity in the exact EF processes measured by these previous studies, we examine three EF factors to determine whether task-positive-to-task-negative network connectivity is linked to EF mechanisms that are common to many tasks (Common EF) or to a more specific EF ability such as Updating-specific or Shifting-specific abilities. Specifically, we test for connectivity-EF relationships in six models predicting each of six pairwise relationships between the frontoparietal, dorsal attention, ventral attention, and default networks from three EF factors, reporting only those relationships that withstand correction for the six models.

One advantage of the current study is that it used a larger sample size (*N* = 250) than typically employed in prior studies of this nature. This approach afforded us the opportunity to investigate two supplemental research questions regarding connectivity-EF relationships that might not have emerged in previous studies of small samples and single EF measures. First, does high Common EF, Shifting-specific, or Updating-specific ability relate to hyperconnectivity (increased positive/decreased negative connectivity) between systems with complementary functions such as the frontoparietal and the dorsal/ventral attention networks or between the dorsal and ventral attention networks themselves? This question is motivated by a finding that stronger positive connectivity between dorsal attention and frontoparietal networks is associated with higher performance on the stop signal task, which is typically considered a measure of inhibitory control (Tian et al., 2013), as well as evidence that task-positive regions become and stay hyperconnected after a challenging EF task (Gordon et al., 2012). Second, is hypo- or hyperconnectivity between lower-level sensory and higher-level cognitive networks related to individual differences in EFs? This question is motivated by prior resting-state work from our group in a younger and smaller sample in which we found Common EF and Shifting-specific ability was linked to the functional connectivity characteristics of lower-level sensory areas (Reineberg and Banich, 2016). Finally, we follow up our primary analyses using a finer-grained network parcellation (*n* = 17) to test spatial specificity (e.g., are brain-EF relationships isolated to particular subcomponents of task-positive networks versus the network at coarse level of analysis?).

## Materials and Methods

### Participants

Participants were 250 individuals from the ongoing Colorado Longitudinal Twin Study [LTS; *M*_(age)_ = 28.7 years, *SD*_(age)_ = 0.57 years; 97 males], who completed a resting state scan as part of a larger testing session. Data from an additional 15 participants were excluded, because they showed excessive levels of movement during the scanning session based on the criteria of greater than 2 mm translation (motion in *X, Y*, or *Z* plane) or 2 degrees rotation (roll, pitch, or yaw motion) (*n* = 14), and failure of the presentation computer to display a fixation cross during the resting scan (*n* = 1). Of the 250 individuals, there were 54 pairs of monozygotic (MZ; identical) twins, 45 pairs of same-sex dizygotic (DZ; fraternal) twins, 24 MZ twin singletons, and 28 DZ twin singletons. Singletons are members of twin pairs whose co-twins either did not participate or were excluded from analysis. All LTS participants were recruited from the Colorado Twin Registry based on birth records, and is representative of the Colorado population at the time of recruitment (see Rhea et al., 2006, 2013 for additional details). Based on self-report, the LTS sample is 92.6% White, 5.0% “more than one race,” <1% American Indian/Alaskan Native, <1% Pacific Islander; 1.2% did not report race. Hispanic individuals composed 9.1% of the sample. Participants were paid $150 for participation in the 3-h study; those who did not finish the entire protocol were paid $25 per half hour. All study procedures were approved by the Institutional Review Board of the University of Colorado Boulder.

### Procedure

The study session involved the administration of behavioral tasks that measured EF ability as well as acquisition of anatomical and functional brain data via MRI. Testing took place in a single 3-h session. Following informed consent, participants were familiarized with the imaging procedures including practice versions of the behavioral tasks to ensure comprehension later in the scanner. They also completed some interviews and questionnaires, then completed a 1.5-h scanning session that began with a structural scan followed by a 6-min resting state, three EF tasks (antisaccade, keep track, and number–letter, in that order), and a diffusion tensor imaging sequence (not analyzed here). The current study only utilizes behavioral data (i.e., reaction time, accuracy) acquired during functional scanning of the antisaccade, keep track, and number-letter tasks. After the scan, participants returned to a behavioral testing room to complete three additional EF tasks (Stroop, category-switch, and letter memory, in that order). If both twins of a pair participated on the same day, the twins completed the protocol sequentially (twin order randomized) with the same ordering of behavioral testing and imaging acquisition.

### Brain Imaging

Participants were scanned in a Siemens Tim Trio 3T scanner. Neuroanatomical data were acquired with T1-weighted MP-RAGE sequence [acquisition parameters: repetition time (TR) = 2400 ms, echo time (TE) = 2.07, matrix size = 320 × 320 × 224, voxel size = 0.80 mm × 0.80 mm × 0.80 mm, flip angle (FA) = 8.00 deg., slice thickness = 0.80 mm]. Resting state data was acquired with a T2^∗^-weighted echo-planar functional scan [acquisition parameters: number of volumes = 816, TR = 460 ms, TE = 27.2 ms, matrix size = 82 × 82 × 56, voxel size = 3.02 mm × 3.02 mm × 3.00 mm, FA = 44.0 deg., slice thickness = 3.00 mm, field of view (FOV) = 248 mm]. During the resting-state scan, participants were instructed to relax and stare at a fixation cross while blinking as they normally would. We based this decision on suggestions in the literature indicating that eyes open and fixated is the optimal instruction for maximizing reliability (Zou et al., 2015). In addition, this approach is thought to minimize the variability that is observed in the visual processing stream when participants are instructed to keep their eyes closed versus open during the resting scan. Visual network variability seemingly comes from top-down imagination/visualization processes, although the exact mechanism is unknown (Patriat et al., 2013).

### Measures

A strength of the LTS sample is a detailed characterization of EF ability. Specifically, rather than measuring EFs with only a single task, we calculated EF factor scores from the six tasks completed on the day of the scan.

#### Antisaccade Task

This task was adapted for fMRI from Roberts et al. (1994). Only behavioral performance was analyzed for the current manuscript. Antisaccade captures the ability to maintain and execute a task set in the face of distracting information; specifically, it requires inhibiting prepotent eye movements (Miyake et al., 2000). In the scanner version, participants completed 20 s blocks of prosaccade, antisaccade, and rest (fixation) trials (12 blocks of each across two runs; 5 trials per block for the prosaccade and antisaccade blocks), each was preceded by a jittered instruction (TOWARD, AWAY, or FIXATION for 2, 4, or 6 s). On each trial, after a jittered fixation lasting 1–3 s, a small visual cue flashed on one side of the computer screen for 234 ms, followed by a target (a digit from 0 to 9) that appeared for 150 ms before being masked. The mask lasted 1650 ms, during which time the participant vocalized the target. The cue and target appeared on the same side of the screen during prosaccade trials and opposite sides during anti-saccade trials. Hence, in order to identify the number on the antisaccade trials, participants had to avoid the automatic tendency to saccade to the cue and instead immediately look in the opposite direction. The dependent measure was the proportion of correctly identified targets on the 60 anti-saccade trials.

#### Stroop Task

This task was adapted from Stroop (1935). Stroop captures the ability to maintain a task set in the face of pre-potent distracting information, specifically, inhibiting the prepotent tendency to read words. Participants verbally indicated the font color (red, blue, or green) of text presented on a black screen as quickly as possible, with reaction time measured via a ms-accurate voice key. Trials were divided up into three types: a block of 42 neutral trials consisting of asterisks (3–5 characters long) presented in one of three colors (red, blue, and green); a block of 42 congruent trials consisting of color words that matched the font color (e.g., the word “RED” displayed in red font); and two blocks of 42 trials each of incongruent trials consisting of color words that did not match the font color (e.g., the word “RED” displayed in blue ink). Each word disappeared as soon as the voice key detected the response, and the next word appeared after a 250 ms white fixation. The dependent measure was the mean reaction time difference between correct incongruent and neutral trials.

#### Keep Track Task

This task was adapted for fMRI from Yntema (1963). Only behavioral performance was analyzed for the current manuscript. Keep track captures the ability to maintain and update information in working memory. On each trial in the scanner version, participants were given 3 or 4 target categories (animals, colors, countries, distances, metals, or relatives) that remained on the screen throughout the trial. After viewing a serial list of 16 words drawn from 6 categories (one word every 2 s), they saw a “???” prompt on the screen for 10 s, during which they orally recalled the last exemplar of each target category. Because each list contained 1–3 exemplars of each category, they had to update which words to remember and ignore words from irrelevant categories. In addition to these “Remember” trials, the scanner version of the task included baseline conditions of “Read” trials, in which participants just silently read the words without trying to remember them, and 20 s rest (fixation) trials. Each trial type was preceded by a jittered instruction (REMEMBER, READ, or FIXATION for 2, 4, or 6 s). There were three runs, each with 3 recall trials (two with 4 words to recall and one with 3), 3 read trials, and 3 rest trials. The behavioral dependent measure was the proportion of the 45 words correctly recalled out of all remember trials.

#### Letter Memory

This task was adapted from Morris and Jones (1990). Letter memory captures the ability to maintain and update items in working memory. In each trial, participants saw a series of 9, 11, or 13 consonants, with each letter appearing for 3 s. As each letter appeared, they had to say aloud the last four letters, including the current letter. The dependent measure was the proportion of 132 sets correctly rehearsed (i.e., the last 4 letters reported in the correct order) across 12 trials.

#### Number–Letter Task

This task was adapted for fMRI from Rogers and Monsell (1995). Only behavioral performance was analyzed for the current manuscript. Number–letter captures the ability to shift between mental sets. In each trial of the scanner version, participants saw a box sectioned into four quadrants. The borders of one quadrant were darkened (i.e., cued) for 350 ms, then a number–letter or letter–number pair (e.g., 4K) appeared inside until it was categorized. The participant had to categorize the number (top 2 quadrants) or letter (bottom 2 quadrants) as odd/even or consonant/vowel, respectively, using two buttons on a button box. The stimuli disappeared from the screen when categorized, and there was a 350 ms response-to-cue interval. The trials were arranged in blocks, and rest blocks (20 s) were intermixed with the task blocks. Each block was preceded by a jittered instruction (TOP, BOTTOM, MIXED, or FIXATION for 2, 4, or 6 s) that indicated where the stimuli would appear for that block. In mixed blocks, half the trials were repeat trials in which the task stayed the same as the previous trial; the other trials required a switch in categorization task. Each block consisted of 13 trials, with the first trial not counted because it was neither switch nor repeat. There were two runs, each containing eight mixed blocks, eight single-task blocks (four each number and letter blocks), and rest blocks. The behavioral dependent measure was the local switch cost — the difference between average response times on correct switch and no-switch trials within mixed blocks (96 trials of each type).

#### Category-Switch Task

This task was adapted from Mayr and Kliegl (2000). Category-switch captures the ability to shift between mental sets. In each trial, participants categorized a word according to animacy (i.e., living vs. non-living) or size (i.e., smaller or larger than a soccer ball), depending on a cue (heart or crossed arrows, respectively) that preceded the word by 350 ms and remained above the word until the participant responded with one of two buttons on a button box. The stimuli disappeared from the screen when categorized, and there was a 350 ms response-to-cue interval. A 200-ms buzz sounded for errors. The task began with two single-task blocks of 32 trials each, in which participants categorized words only by animacy then only by size. Then participants completed two mixed blocks of 64 trials each, in which half the trials required switching the categorization criterion. The dependent measure was the local switch cost — the difference between average response times on correct switch and no-switch trials within mixed blocks.

### Data Analysis

#### EF Data

Scores on the six EF tasks were subjected to the same trimming and transformation used in prior studies to improve normality and reliability (Friedman et al., 2016). Specifically, correct reaction times were trimmed within-subject to obtain the best measures of central tendency within conditions (Wilcox and Keselman, 2003). Additionally, within the number-letter and category-switch tasks, trials following error trials were excluded, as determining switch versus repeat trials is dependent on the preceding trial. Following within-subject reaction time trimming, extreme high and low scores at the between-subjects level (greater than 3 *SDs* from the group mean) were Windsorized (replaced with the cutoff value of 3 *SD*s above or below the mean, respectively) to improve normality and reduce the impact of extreme scores while maintaining these scores in the distribution.

Factor scores were extracted via a confirmatory factor analysis in Mplus 8.0 (Muthén and Muthén, Muthén and Muthén), with all six EF tasks loading on Common EF, the keep track and letter memory tasks loading on the orthogonal Updating-specific factor, and the number–letter and category-switch tasks loading on the orthogonal Shifting-specific factor. The loadings were equated (after scaling the measures to have similar variances) within the Updating-specific and Shifting-specific factors to identify these two-indicator factors.

#### Preprocessing

All processing of brain data was performed in a standard install of FSL build 5.09 (Jenkinson et al., 2012). To account for signal stabilization, the first 10 volumes of each individual functional scan were removed, yielding 806 volumes per subject for additional analysis. The functional scans were corrected for head motion using MCFLIRT, FSL’s motion correction tool. Brain extraction (BET) was used to remove signal associated with non-brain material (e.g., skull, sinuses, etc.). FSL’s FLIRT utility was used to perform a boundary-based registration of each participant’s functional scan to his or her anatomical volume and a six-degree-of-freedom affine registration to MNI152 standard space. LTS scans were subjected to AROMA, an automated independent components analysis-based, single-subject de-noising procedure (Pruim et al., 2015). Signal was extracted from masks of the lateral ventricles, white matter, and whole brain volume and regressed out along with a set of six motion regressors and associated first and second derivatives. The scans were band-pass filtered (0.001–0.08 Hz band). Finally, time courses for each of the functional networks of interest were extracted for each individual with FSL’s “fslmeants” command (Jenkinson et al., 2012) using the network templates provided by Yeo and colleagues as a mask.

#### Statistical Models

We used the time courses generated by the procedure outlined above to determine whether or not individual differences in network-to-network connectivity are associated with variation in EF ability. We calculated network-to-network connectivity as Fisher’s *z*-corrected Pearson’s *r*-values for all pairwise relationships between functional networks of interest. We then performed a multiple regression analysis regressing network-to-network connectivity on Common EF, Shifting-specific, and Updating-specific factor scores as well as gender and mean translation and rotation movement during the resting-state scan. To account for non-independence of twin pairs, we utilized the “type = complex” option in Mplus. This option uses a sandwich estimator to obtain standard errors corrected for familial clustering. The relevant measures were treated as approximately continuous variables using the robust maximum likelihood (MLR) estimator.

Because we had genetically informative data, we evaluated whether significant associations were present within-families and/or between-families, using a multilevel twin difference model (Vitaro et al., 2009). Specifically, we used a random intercepts model of the connection strength, with level 1 (within-family) predictors of each twin’s deviation from his or her family mean of Common EF, Shifting-specific, and Updating-specific score (i.e., cluster-centered), as well as grand-mean-centered translation and rotation. The slopes for the within-family Common EF, Shifting-specific, and Updating-specific effects were allowed to vary by zygosity, but were not allowed to have residual variance (i.e., we specified these slopes as random, regressed them on zygosity at level 2, and fixed their residual variances to zero). At level 2 (between), we regressed the random intercept on the family means for Common EF, Shifting-specific, and Updating-specific scores, as well as sex (which did not vary within families). We standardized all continuous variables to obtain parameter estimates in standard deviation units. The Mplus syntax for this model is provided in the Supplementary Material.

## Results

### Behavioral Data

Descriptive statistics for all behavioral tasks are provided in **Table 1A**, while the factor scores for Common EF, Shifting-specific EF, and Updating-specific EF are provided in **Table 1B**. In latent variable form, Common EF, Shifting-specific, and Updating-specific are orthogonal; however, their factor scores are moderately correlated because they are imperfect approximations of latent variables (factor score indeterminacy). Factor score determinacy estimates for the complete data pattern were 0.83, 0.75, and 0.60 for Common EF, Shifting-specific EF, and Updating-specific EF, respectively. Common EF was positively correlated with Updating-specific EF (*r* = 0.33, *p* < 0.001) and Shifting-specific EF (*r* = 0.20, *p* < 0.001), whereas Updating-specific EF and Shifting-specific EF were negatively correlated (*r* = -0.33, *p* < 0.001).

**Table 1:** Descriptive statistics for executive function tasks, measures, and correlations among resting-state networks.

		Descriptive statistics						
		Mean	Std	Minimum	Maximum	Skewness	Kurtosis	Reliability
**(A)**	Antisaccade	43.87%	21.35	5.00	96.67	0.37	-0.67	0.90*
	Stroop	154.44 ms	77.34	-3.14	395.60	0.81	0.67	0.96*
	Keep track	75.63%	14.12	34.22	100.00	-0.66	0.03	0.74^∧^
	Letter memory	71.48%	14.01	35.61	100.00	0.06	-0.87	0.93^∧^
	Number-letter	171.12 ms	106.01	-41.36	508.88	0.84	0.89	0.81*
	Category switch	203.79 ms	175.29	-64.78	744.85	1.33	1.49	0.94*
		**Mean**	**Std**	**Minimum**	**Maximum**	**Skewness**	**Kurtosis**	
**(B)**	Common EF	0.017	0.830	-2.202	2.083	0.027	-0.415	
	Shifting-specific	-0.015	0.748	-2.571	1.566	-0.717	0.359	
	Updating-specific	0.011	0.602	-1.938	1.624	-0.343	0.092	
		**Mean**	**Std**	**Minimum**	**Maximum**	**Skewness**	**Kurtosis**	
**(C)**	V_to_SM	-0.224	0.341	-1.172	0.844	0.215	0.229	
	V_to_DAN	0.254	0.310	-0.529	1.017	0.010	-0.560	
	V_to_VAN	-0.092	0.359	-1.173	0.729	-0.333	0.086	
	V_to_L	-0.372	0.293	-1.364	0.492	-0.200	0.322	
	V_to_FP	-0.431	0.289	-1.280	0.265	-0.243	-0.101	
	V_to_DEF	-0.496	0.287	-1.202	0.365	-0.038	-0.075	
	SM_to_DAN	-0.054	0.303	-1.260	0.769	-0.304	0.483	
	SM_to_VAN	0.407	0.337	-0.733	1.266	-0.019	0.603	
	SM_to_L	-0.101	0.314	-0.948	0.720	-0.152	-0.126	
	SM_to_FP	-0.387	0.296	-1.111	0.348	0.021	-0.349	
	SM_to_DEF	-0.227	0.301	-0.943	0.726	0.196	-0.196	
	DAN_to_VAN	0.360	0.335	-0.635	1.293	-0.249	0.264	
	DAN_to_L	-0.393	0.292	-1.140	0.515	0.130	-0.022	
	DAN_to_FP	0.144	0.327	-0.663	0.968	-0.104	-0.471	
	DAN_to_DEF	-0.917	0.289	-1.628	-0.030	0.369	0.006	
	VAN_to_L	-0.323	0.303	-1.059	0.553	0.246	-0.346	
	VAN_to_FP	0.069	0.321	-0.737	0.976	-0.002	-0.170	
	VAN_to_DEF	-0.823	0.332	-1.663	0.360	0.397	0.184	
	L_to_FP	-0.096	0.336	-1.024	0.833	-0.032	-0.333	
	L_to_DEF	0.614	0.331	-0.318	1.687	-0.030	-0.158	
	FP to DEF	-0.020	0.332	-0.875	0.979	0.131	-0.243	

***(A)***Descriptive statistics for six EF tasks.****(B)****Descriptive statistics for Common EF, Shifting-specific EF, and Updating-specific EF.****(C)****Descriptive statistics for correlations amongst seven resting-state networks. V, visual network; SM, sensory/somatomotor network; DAN, dorsal attention network; VAN, ventral attention network; FP, frontoparietal network; DEF, default network. For example, the mean for V_to_SM is Fishers’* z-transformation of Pearson’s correlation between visual and sensory/somatomotor networks. ^∗^Split-half reliability (odd/even for Stroop and Category-switch or run1/run2 for antisaccade and number–letter), adjusted with the Spearman-Brown prophecy formula. ^∧^Chronbach’s alpha across 3 runs for keep track and 4 sets of trials for letter memory*.

### Mean Network Connectivity

**FIGURE 1:**
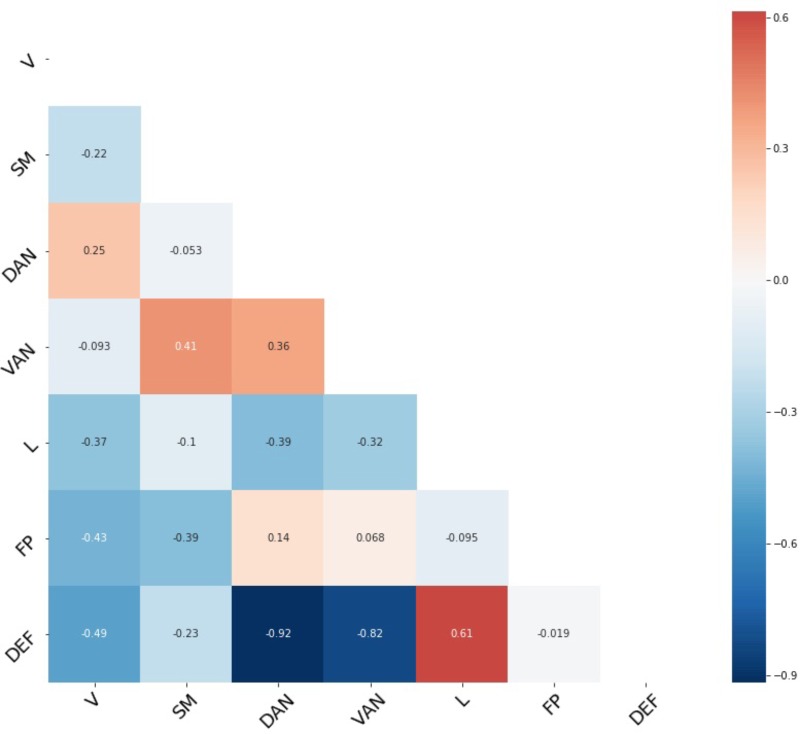
Average network connectivity. Fisher’s *z*-transformation of Pearson’s correlation between each pair of seven functional networks. V, visual network; SM, sensory/somatomotor network; DAN, dorsal attention network; VAN, ventral attention network; FP, frontoparietal network; DEF, default network.

Average connectivity among all seven functional networks provides some assurance the current sample is consistent with prior work and serves as a validity check. **Figure 1** shows all group average pairwise correlations between each of the seven functional networks, while descriptive statistics for all network-to-network connectivity measures are provided in **Table 1C**. As expected, there is an average positive connectivity between dorsal and ventral attention networks, and an average negative connectivity between the default network (i.e., implicated in internally directed attention) and attention networks (i.e., implicated in external attention). However, the relationship between the frontoparietal and default network was slightly positive, on average.

### Relationship Between Network Connectivity and EF

#### Analysis of Higher-Level Cognitive Networks – General

**FIGURE 2:**
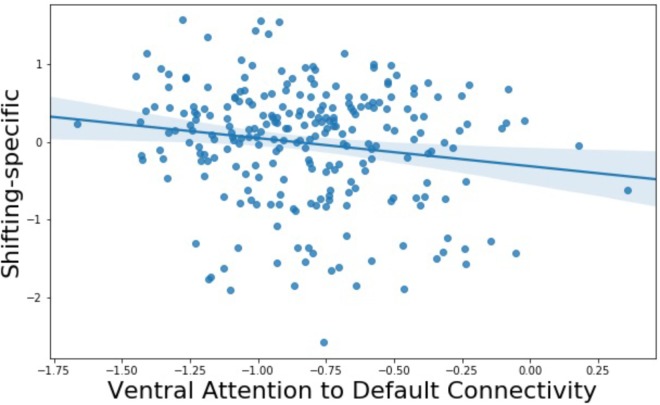
Relationship between Shifting-specific factor scores and ventral attention-to-default network connectivity.

The primary analyses used to investigate network connectivity and individuals differences in levels of EF were six multiple regression models in which each pairwise connection between the default, frontoparietal, dorsal attention, and ventral attention networks was regressed on the Common EF, Shifting-specific, and Updating-specific factor scores while controlling for a summary of motion during the resting-state scan and gender. After Bonferroni correcting for these six models (alpha = 0.05/6 = 0.0083), we found one EF parameter estimate was statistically significant: Individuals with higher Shifting-specific scores had reduced connectivity between the ventral attention and default networks (**Figure 2**; standardized beta = -0.181, *p* = 0.005). This particular connection was strongly negatively correlated across the group, so, individuals with better Shifting-specific ability had stronger negative correlations between these two systems.

**FIGURE 3:**
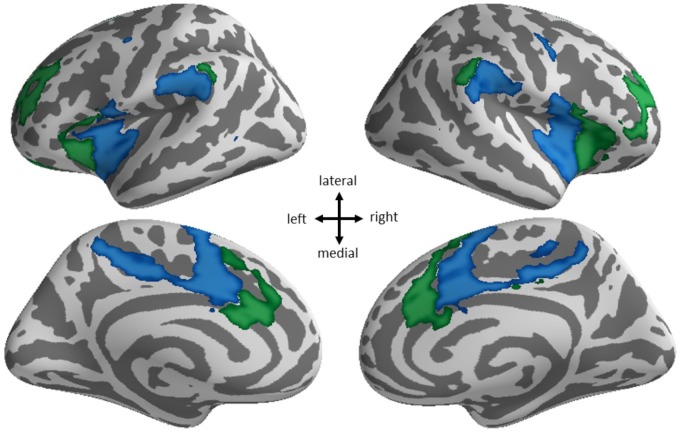
Spatial specificity of ventral attention network subsystems. The ventral attention network from the 7-network parcellation (blue + green) breaks into two subsystems in the 17-network parcellation: anterior (green) and posterior (blue).

**FIGURE 4:**
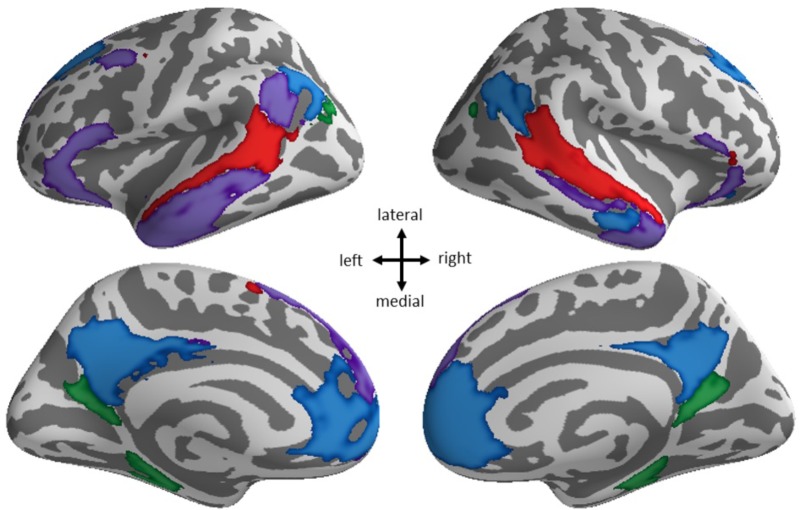
Spatial specificity of default network subsystems. The default network from the 7-network parcellation (blue + purple + red + green) breaks down into four subsystems in the 17-network parcellation: hub subsystem (blue), superior and lateral frontal/inferior temporal cortex subsystem (purple), superior temporal lobe subsystem (red), and posterior cingulate/precuneal (green) subsystems.

To further explore this finding, we investigated the spatial specificity of the connectivity between the ventral attention network and the default network as it relates to Shifting-specific EF, by using the multiple ventral attention and default network subcomponents from Yeo et al. (2011) 17-network parcellation. This parcellation divides the ventral attention network into two subcomponents that are primary differentiated by involving anterior as compared to posterior divisions of all the key cingulate, insular, and temporal/parietal areas. The default network is divided into four subcomponents: three are divisions of the midline hubs and lateral parietal aspects of the default network, and another is best described as the temporal lobe subsystem of the default network. Our supplemental analysis found higher Shifting-specific was associated with more negative connectivity of the posterior ventral attention subsystem (**Figure 3**, blue) and the hub subsystems of the default network (**Figure 4**, blue), but notably not the temporal lobe subsystem of the default network.

Additionally we considered some other aspects of our findings. While statistically significant but not passing correction for multiple comparisons we found that individuals with higher Shifting-specific scores had reduced connectivity between frontoparietal and ventral attention networks (standardized beta = -0.159, *p* = 0.032), See **Table 2A** for standardized beta weights for all models of *a priori* interest.

**Table 2:** Standardized beta weights for models predicting network-to-network connectivity from three EF factor scores.

		Beta (CEF)	Beta (SHI)	Beta (UPD)
**(A)**	DAN_to_VAN	-0.124	0.092	0.037
	DAN_to_FP	0.032	-0.079	0.021
	DAN_to_DEF	0.023	-0.050	-0.117
	VAN_to_FP	0.140	-0.159*	-0.073
	VAN_to_DEF	0.067	-0.181***	-0.047
	FP_to_DEF	-0.110	0.009	-0.056
		**Beta (CEF)**	**Beta (SHI)**	**Beta (UPD)**
**(B)**	V_to_DAN	0.066	0.173**	0.144
	V_to_VAN	-0.056	0.176**	0.085
	V_to_FP	0.007	0.172*	0.115
	V_to_DEF	-0.040	-0.051	-0.054
	SM_to_DAN	-0.171*	0.035	0.021
	SM_to_VAN	-0.015	-0.006	-0.071
	SM_to_FP	-0.117	0.071	0.054
	SM_to_DEF	0.203**	-0.043	-0.076

***(A)***Results from 6 models involving higher-level cognitive networks of a priori interest.****(B)****Results from 8 models involving connectivity between higher-level cognitive and visual/sensory-somatomotor networks. V, visual network; SM, sensory/somatomotor network; DAN, dorsal attention network; VAN, ventral attention network; FP, frontoparietal network; DEF, default network. ^∗^p < 0.05, ^∗∗^p < 0.01, ^∗∗∗^p < Bonferroni corrected alpha [0.0083 for****(A)****and 0.0036for****(B)***]*.

Moreover, due to multiple reports of frontoparietal-to-default hypoconnectivity being significantly associated with working memory span/sequencing, we specifically interrogated this relationship. Although the direction of the relationship for Common EF was consistent with these prior reports, such that higher Common EF scores were associated with hypoconnected frontoparietal and default networks, the effect was not significant (standardized beta = -0.110, *p* = 0.142).

#### Analysis of Higher-Level Cognitive Networks – Genetic Influences

Because we had genetically informative data, we evaluated whether the significant association between Shifting-specific ability and the connectivity of ventral attention and default networks was influenced by genetic factors. To do so, we used a multilevel twin difference model (Vitaro et al., 2009). If the effect is significant within MZ twin pairs (i.e., the twin with the higher Shifting-specific score has a more negative connection between ventral attention and default networks), it suggests the effect is due to non-shared environmental influences that affect both connection strength and Shifting-specific ability. Such a finding would be consistent with, but not assuring of, a causal effect. A between-family effect suggests that differences between families (which can include genetic and shared environmental effects such as socioeconomic status) drive the association. We found a significant between-family effect (beta = -0.159, *p* = 0.022) of Shifting-specific EF on the connection strength between the ventral attention and default networks. The within-family effect was not significant averaging across zygosity (beta = -0.126, *p* = 0.303), but there was a marginally significant interaction of the within effect by zygosity (beta = 0.468, *p* = 0.053), such that there is a marginally significant within effect for MZ pairs (simple effect beta = -0.354, *p* = 0.055) but not DZ pairs (simple effect beta = 0.114, *p* = 0.469). Together, these effects are evidence suggesting genes and shared environments influence the relationship between Shifting-specific EF and connectivity and preliminary evidence of non-shared environmental influences.

#### Exploratory Analysis of Sensory Networks

Our final analyses explored associations of connectivity between higher-level and lower-level systems and Common EF, Shifting-specific EF, and Updating-specific EF. Specifically, we used the same multiple regression models described above to predict pairwise connections between the higher-level cognitive networks discussed above and the visual and somatomotor networks, respectively. We found higher Shifting-specific was associated with greater positivity connectivity between the visual network and the frontoparietal network (standardized beta = 0.172, *p* = 0.017), the visual network and dorsal attention network (standardized beta = 0.173, *p* = 0.010), and the visual network and ventral attention network (standardized beta = 0.176, *p* = 0.007). Hence, higher Shifting-specific EF is associated with greater positive connectivity between the visual network and higher-order executive/attention networks. In addition, higher Common EF was associated with a more negative relationship between activity in the somatomotor and dorsal attention network (standardized beta = -0.171, *p* = 0.014) and with a more positive relationship between the somatomotor and default network activity (standardized beta = 0.203, *p* = 0.005). However, no exploratory results were significant after Bonferroni correction for the six original and eight additional models (alpha = 0.05/14 = 0.0036). See **Table 2B** for standardized beta weights for all models of exploratory interest.

## Discussion

We investigated the associations between three EF components and coordination among large scale brain systems, with a particular focus on brain systems involved in higher-level cognition. We found that better abilities specific to quickly shifting between task sets, as measured by a Shifting-specific factor score, are related to more negative connectivity between a brain system involved in internal mentation (the default network) and the ventral attention network. We will first discuss this principal finding in more detail and then discuss this result in the context of prior reports of behavior-related hypoconnectivity of higher-level cognitive networks with default networks in both the clinical domain and in neurologically normal individuals. Finally, we discuss findings of exploratory analyses regarding network connectivity between higher-level cognitive networks and lower-level networks such as the visual network and the somatomotor network.

Our primary analysis revealed a novel relationship between Shifting-specific ability and connectivity between the default and ventral attention networks. A test of spatial specificity further revealed the effect may be primarily driven by connectivity between the midline regions of the default network hubs — ventromedial prefrontal (vmPFC) and posterior cingulate cortices (PCC) — and a posterior subsystem of the ventral attention network.

To put this finding in perspective, we consider the purported functions of these regions. A review of the functions of the default network suggests that the midline hubs of the default network are involved in many aspects of self-referential processing including self-reflection, mentalizing, autobiographical memory, and episodic future thinking among others (Andrews-Hanna, 2011).

The ventral attention network, in the context of the Yeo et al. (2011) parcellation, contains at least three main subsystems: higher-level visual/attention areas (temporo-parietal junction), right lateral prefrontal cortex, and the cingulo-opercular system (predominantly insula and dorsal anterior cingulate cortex). One popular theory of the function of the ventral attention system suggests this part of cortex specializes in detection of behaviorally relevant stimuli (Corbetta and Shulman, 2002) and reorientation of attention toward relevant environmental information (Vossel et al., 2014). However, our examination of spatial specificity of the effect we observed using a finer-grained network parcellation revealed that although Shifting-specific EF was related to connectivity of the ventral attention network as a whole, the effect may be driven more specifically by connectivity of the cingulo-opercular subsystem. Characterization of the functions of the cingulo-opercular system is a topic of considerable interest and current controversy. The cingulo-opercular system has unique cytoarchitectonic properties (Seeley et al., 2012) and is often implicated in very broad cognitive constructs such as alertness, maintenance, and awareness (Dosenbach et al., 2007; Craig, 2009; Craig, 2011; Sadaghiani and D’Esposito, 2015; Coste and Kleinschmidt, 2016), perhaps in part due to insula’s high base rate of activation in fMRI studies (Yarkoni et al., 2011). A detailed functional description of anterior and posterior subsystems of the ventral attention network does not currently exist. However, a meta-analysis of the insula using thousands of fMRI studies as ascertained from Neurosynth (Yarkoni et al., 2011) revealed the posterior portion of the insula identified in the current report may be functionally distinguished from anterior portions by processing related to switching, inhibition, error processing, conflict, feedback, somatosensory, and other terms (Chang et al., 2013). That is, although anterior and posterior insula are involved in very similar types of processing, the posterior region may be activated more than anterior portions in certain EF-related contexts (i.e., switching, inhibition, etc.). Regarding EFs more directly, it has been proposed that the insula may play a critical role in regulating the coordination of frontoparietal and default network functions (Sridharan et al., 2008; Goulden et al., 2014). Work in the clinical domain supports the notion that ventral attention network functioning is compromised in disorders that often have comorbid EF deficits, such as anxiety (Sylvester et al., 2012) and depression (Kaiser et al., 2016).

Considering the functions of the default and ventral attention networks, one must ask how coordination of the default and ventral attention networks translates to increased performance in a specific aspect of EF that involves the rapid/fluid shifting between task/mental sets and rules, over and above goal maintenance or other general EF abilities (Common EF). Intrinsic network connections in high shifting ability individuals could be a specific, optimized state that places that individual metabolically closer to the brain states required when performing difficult cognitive tasks. Prior work has shown that better performers in a variety of cognitive domains have smaller changes in functional connectivity when going from rest to a task-directed state, possibly reflecting more efficient neural configurations (Schultz and Cole, 2016). In the context of the current study, perhaps more negative default to ventral attention connectivity is a brain state uniquely beneficial for shifting functions. From the perspective that stable resting-state connectivity reflects a history of co-activation (Wig et al., 2011), better shifters may have a stronger history of suppressing default network activity during times when interference from internal mentation functions may be disadvantageous (for a review of default network deactivation and hypoconnectivity see Anticevic et al., 2012), for example, when mind wandering might be detrimental to performance on a demanding task (Weissman et al., 2006). However, the exact mechanism through which network connectivity translates to increased performance is still an open question.

Our study also provided an example of how genetically informative data can be used to provide insights about the causes of inter-individual variation in network connectivity. Prior work utilizing a large sample of twins revealed the cross-twin correlation of default-to-cingulo-opercular connectivity was moderate and significant for both MZ (*r* = 0.336) and DZ (*r* = 0.245) twins, stronger for MZ twins, and substantially lower than 1 (Yang et al., 2016). This pattern of results indicates mixed influences of genes, shared environments and non-shared environments. Although a classic twin model to estimate the genetic, shared environmental, and non-shared environmental influence on ventral attention-to-default connectivity could be applied to the data in current study, due to small sample size we opted to perform a multilevel twin difference model. This analysis revealed that the ventral attention-to-default network connectivity relationship with Shifting-specific EF is primarily driven by between-family differences, which include both genetic and shared environmental influences. We also observed a marginally significant within-family effect for MZ twins (but not DZ twins), which suggests the non-shared environmental influences that cause one MZ twin to have higher Shifting-specific ability than his or her co-twin may be the same non-shared environmental influences that cause that MZ twin to have more negative ventral attention-to-default connectivity. Future work using larger twin samples should continue this line of research to tease apart genetic and environmental influences on network and regional connectivity.

Regarding other associations between higher-level cognitive network connectivity and EFs, we did not find any other strong associations after correcting for multiple comparisons. Nonetheless, there were some results worth noting. First, we did find that individuals with higher Shifting-specific scores had increased negative connectivity between the frontoparietal and ventral attention systems, which reached a univariate level of significance (*p* < 0.05) but did not when Bonferroni-corrected. At first glance, increased Shifting-Specific EF ability and a reliance upon non-simultaneous activation of two closely related systems might seem counterintuitive, as one might have expected higher EF ability to be associated with greater co-activation of closely related, higher-level cognitive systems. However, prior work has established that EF requires a trade-off between cognitive stability and flexibility, with stability required to impose and maintain a task set, and flexibility required to switch between tasks and subgoals (Goschke, 2000) with flexibility-related measures (such as Shifting-specific EF) sometimes showing the opposite relationship with outcomes than measures of stability (see Herd et al., 2014; Friedman and Miyake, 2017). Examples of such findings are studies that found a relationship between increased shifting-specific ability and increased substance use (Gustavson et al., 2017), decreased intelligence, and poorer self-restraint (Friedman et al., 2011). Although speculative, perhaps this brain-behavior relationship is a neural manifestations of the flexibility-stability tradeoff.

Based on prior findings in the clinical domain and limited work with neurologically normal individuals (e.g., Kelly et al., 2008), we expected to find that higher Common EF or Updating-specific EF would be associated with reduced connectivity between the frontoparietal and default networks. We did not find this result. However, we did find a trend for individuals with higher Common EF to have a more negative relationship between activation in the frontoparietal and default networks, consistent with expectations. Although our results suggest there is no reliable association between default-to-frontoparietal network connectivity and EFs, we did not test for association between EFs and connectivity at the level of small and specific regions-of-interest (as in Keller et al., 2015) or between larger conglomerate networks that might combine signal across many task-positive networks (e.g., frontoparietal, dorsal attention, and cingulo-opercular regions; as in Kelly et al., 2008). In summary, the results of the current study complement prior research in the area of EF-connectivity relationships by providing an alternative measurement of both EF behavior (i.e., in the context of the Unity/Diversity model) and connectivity at the level of seven functional networks.

In an exploratory analysis, we investigated associations between EFs and connectivity between higher-level cognitive and lower-level sensory networks. We found higher Shifting-specific EF was associated with increased positive connectivity between the visual network and each of the task-positive networks (frontoparietal, dorsal attention, and ventral attention). These results are novel but complement prior work from our group in a younger sample in which we showed individuals with higher Shifting-specific ability had more diffusely connectivity visual cortices as quantified by local clustering coefficient, a graph theoretic measure (Reineberg and Banich, 2016). We also found higher Common EF was associated with more positive connectivity between the somatomotor and default networks as well as more negative connectivity between the somatomotor and dorsal attention networks. These findings are both novel and should be replicated/explored in future work. Generally, the results of our exploratory analysis suggest EFs may rely on broad patterns of connectivity across many brain systems, including those that are not typically associated with inter-individual variation on EF tasks (e.g., visual network and sensory/somatomotor network).

It is important to consider some limitations of the current work. As alluded to earlier, the mechanism through which network connectivity influences behavior is unclear. The stability of resting-state measures suggests high-EF individuals may have intrinsic brain characteristics that foster or allow for their higher behavioral performance. But in contrast, a substantial literature shows the malleability of connectivity in the face of specific cognitive challenges and state inductions (Spreng et al., 2010; Fornitoa et al., 2012; Cocchi et al., 2013). This literature suggests high-EF individuals could be in cognitive states during resting-state scans that differentiate them from low ability individuals – for example, simulating, planning, or rehearsing rules for cognitive tasks that are part of the testing session. Future work could utilize experience sampling or experimental manipulations of task instructions/order to rule out these possible mechanisms. Another limitation of the current study is quantification of resting-state connectivity in a static manner. Dynamic connectivity methods are an alternative that measure changes in network connectivity over the course of a resting-state scan rather than as a single summary of the entire scan (Allen et al., 2014; Dixon et al., 2017). Preliminary work in this area suggests dynamics may be related to individual differences in cognitive abilities (Liu et al., 2017; Nomi et al., 2017), so future work could build upon the current study by measuring these dynamics in relation to multiple EF factors. Finally, although we utilized a large sample for investigating the neural basis of individual differences in EFs, we will be able to more accurately estimate genetic and environmental influences on the behaviorally relevant brain signals described in the current work as we increase the sample size of this ongoing study.

In summary, using a large sample of twins with thoroughly measured EF abilities, we have provided a new perspective on cognitively relevant signals in the connectome, particularly connectivity among large scale networks with broadly defined higher-level cognitive functions. Our work suggests “hypoconnectivity” or “anticorrelation” of functional networks may be an important indicator of skill/ability. Individuals who are better able to fluidly shift between mental task sets had more negatively correlated default and ventral attention networks than their less skilled peers.

## Ethics Statement

This study was carried out in accordance with the recommendations of the Institutional Review Board of the University of Colorado Boulder. The protocol was approved by the Institutional Review Board of the University of Colorado Boulder. All subjects gave written informed consent in accordance with the Declaration of Helsinki.

## Author Contributions

MB, NF, and AR designed the research. AR and NF performed the analysis. All authors wrote the manuscript.

## Conflict of Interest Statement

The authors declare that the research was conducted in the absence of any commercial or financial relationships that could be construed as a potential conflict of interest.
