# Supplementary material for: The Relationship Between Resting State Network Connectivity and Individual Differences in Executive Functions
Source: Front Psychol. 2018 Sep 5;9:1600. doi: 10.3389/fpsyg.2018.01600 (PMC6134071; doi:10.3389/fpsyg.2018.01600)
Supplement: Supplementary file 1 [file Data_Sheet_1.DOCX]

**Supporting Information**

Mplus Syntax for Multilevel Twin Model

TITLE: Multilevel model of EFs predicting connection

DATA: FILE IS data.csv;

VARIABLE: NAMES =

snum,twinid,Sex,Trand,Zyg,family,

translation,rotation,

cEF3,SHI3,UPD3,VAN_to_DEF;

MISSING are .;

CLUSTER= Family;

USEVARIABLES = translation rotation sex

cEF3 SHI3 UPD3 ZYG brain

cEF3b SHI3b UPD3b;

CLUSTER = family;

WITHIN = cEF3 UPD3 SHI3 translation rotation;

BETWEEN = cEF3b SHI3b UPD3b ZYG sex;

DEFINE:

brain = VAN_to_DEF;

standardize cEF3 UPD3 SHI3; !get standardized EF factor scores

!get family means for EF factor scores for between regressors

cEF3b = cluster_mean(cEF3);

UPD3b = cluster_mean(UPD3);

SHI3b = cluster_mean(SHI3);

!center slopes so intercepts are for MZ group

ZYG = ZYG -1;

!center EF factor scores within families for within regressors

center cEF3 SHI3 UPD3 (groupmean);

!center/standarize regressors

center sex (grandmean);

standardize brain cEF3b UPD3b SHI3b translation rotation;

ANALYSIS: Type = twolevel RANDOM;

MODEL:

%WITHIN%

!each EF factor score has a random slope factor that can be regressed on zygosity at between level

Sef| BRAIN ON cEF3;

Supd| BRAIN ON UPD3;

Sshi| BRAIN ON SHI3;

BRAIN ON translation rotation; !covarying motion

%BETWEEN%

BRAIN on cEF3b UPD3b SHI3b sex; !regressing random intercept on family means

sef supd sshi ON ZYG; !regressing random slopes for EF factors predicing brain on zygosity

sef@0 supd@0 sshi@0; !no residual variances for slopes

output: samp cint;
